# Acupuncture to improve tolerance of diagnostic esophagogastroduodenoscopy in patients without systemic sedation: results of a single-center, double-blinded, randomized controlled trial (DRKS00000164)

**DOI:** 10.1186/s13063-016-1468-0

**Published:** 2016-07-26

**Authors:** Anja Schaible, Katja Schwan, Thomas Bruckner, Konstanze Plaschke, Markus W. Büchler, Markus Weigand, Peter Sauer, Christian Bopp, Phillip Knebel

**Affiliations:** 1 Department of General, Visceral and Transplantation Surgery, University of Heidelberg, INF 110, 69120 Heidelberg, Germany; 2 Department of Anaesthesiology, GRN-Hospital, Eberbach, Germany; 3 Institute of Medical Biometry and Informatics, University of Heidelberg, Heidelberg, Germany; 4 Department of Anaesthesiology, University of Heidelberg, Heidelberg, Germany; 5 Department of Gastroenterology, University of Heidelberg, Heidelberg, Germany; 6 Department of Anaesthesiology, GRN-Hospital, Schwetzingen, Germany

**Keywords:** Acupuncture, endoscopy, esophagogastroduodenoscopy, sedation

## Abstract

**Background:**

Sedation prior to esophagogastroduodenoscopy is widespread and increases patient comfort. However, it demands additional trained personnel, accounts for up to 40 % of total endoscopy costs and impedes rapid hospital discharge. Most patients lose at least one day of work. 98 % of all serious adverse events occurring during esophagogastroduodenoscopy are ascribed to sedation. Acupuncture is reported to be effective as a supportive intervention for gastrointestinal endoscopy, similar to conventional premedication. We investigated whether acupuncture during elective diagnostic esophagogastroduodenoscopy could increase the comfort of patients refusing systemic sedation.

**Methods:**

We performed a single-center, double-blinded, placebo-controlled superiority trial to compare the success rates of elective diagnostic esophagogastroduodenoscopies using real and placebo acupuncture. All patients aged 18 years or older scheduled for elective, diagnostic esophagogastroduodenoscopy who refused systemic sedation were eligible; 354 patients were randomized. The primary endpoint measure was the rate of successful esophagogastroduodenoscopies. The intervention was real or placebo acupuncture before and during esophagogastroduodenoscopy. Successful esophagogastroduodenoscopy was based on a composite score of patient satisfaction with the procedure on a Likert scale as well as quality of examination, as assessed by the examiner.

**Results:**

From February 2010 to July 2012, 678 patients were screened; 354 were included in the study. Baseline characteristics of the two groups showed a similar distribution in all but one parameter: more current smokers were allocated to the placebo group. The intention-to-treat analysis included 177 randomized patients in each group. Endoscopy could successfully be performed in 130 patients (73.5 %) in the real acupuncture group and 129 patients (72.9 %) in the placebo group. Willingness to repeat the procedure under the same conditions was 86.9 % in the real acupuncture group and 87.6 % in the placebo acupuncture group.

**Conclusions:**

Esophagogastroduodenoscopy without sedation is safe and can successfully be performed in two-thirds of patients. Patients planned for elective esophagogastroduodenoscopy without sedation do not benefit from acupuncture of the Sinarteria respondens (Rs) 24 Chengjiang middle line, Pericard (Pc) 6 Neiguan bilateral, or Dickdarm (IC) 4 Hegu bilateral, according to traditional Chinese medicine meridian theory.

**Trial registration:**

DRKS00000164. Registered on 10 December 2009.

## Background

More than 10 million upper gastrointestinal endoscopic procedures are performed in the United States annually [1, 2], and more than 2.8 million in Germany [3]. The standard use of systemic sedation to facilitate the performance of esophagogastroduodenoscopy and increase patient comfort has contributed to the widespread use and acceptance of this procedure. However, the perceived benefits of improved patient comfort and satisfaction afforded by parenteral sedation must be measured against the increased risk of adverse cardiopulmonary events and higher attendant costs [4].

Complications arising from esophagogastroduodenoscopy are usually associated with the use of systemic sedation and the dose given. More than 60 % of all adverse events [5] and more than 98 % of all serious adverse events in upper gastrointestinal endoscopy are ascribed to systemic sedation [6]. In 2008, the first S3-guidelines for sedation to improve patient safety in gastrointestinal endoscopy in Germany were published [7]. Besides other considerations, sedation demands additional trained personnel. Therefore, it is estimated that sedation and related issues account for up to 40 % of total endoscopy costs, including overhead and indirect costs [8, 9]. Specifically, an additional specialized nurse or physician is required to perform and monitor systemic sedation.

Furthermore, systemic sedation impedes rapid hospital discharge, causing patients to miss work. Following esophagogastroduodenoscopy, the individual may feel well and often believes that he or she has no functional impairment. In contrast, studies of psychomotor effects show that the effects of systemic sedation can extend for 3 to 12 hours from the end of the procedure until patients have recovered clinically [10], and most patients lose at least one day of work [11].

Moreover, especially in the early postoperative period, surgical patients often have impaired gastric function and duodenogastroesophageal reflux, resulting in delayed gastric emptying [12]. Therefore, conscious sedation is often not possible or has high complication rates in these patients, owing to the risk of aspiration.

Acupuncture has been used as a part of traditional Chinese medicine for more than 2000 years [13]. Many studies have investigated the benefits and success of acupuncture in reducing pain for various acute and chronic diseases. However, most of them had methodological difficulties, e.g. the inclusion of an adequate control group [14]. A Cochrane review from 2009 on acupuncture for migraine prophylaxis reported that even acupuncture at the wrong place (sham acupuncture) could have a significant effect on the primary endpoint measure [15]. Therefore, the use of a real placebo needle seems to be a better alternative to overcome this problem. With the introduction of such a placebo acupuncture needle system some years ago, a new and valid instrument to measure placebo effects has become available [16].

If the use of acupuncture could improve examination quality and tolerance of diagnostic esophagogastroduodenoscopy without sedation, it should be possible to increase the willingness of patients to undergo this examination without systemic sedation. Consequently, it would be possible to reduce the rate of serious adverse events due to systemic sedation and to lower the personnel and material costs. In 2004, a review article concluded that acupuncture seems to be effective as a supportive intervention for gastrointestinal endoscopy, providing similar tolerability to that of conventional premedication but superior tolerability to that of sham acupuncture [17].

To date, only one double-blind controlled trial of patients undergoing esophagogastroduodenoscopy has been performed [18]. The study, reported in 1978, used real versus sham acupuncture (1 cm away from the acupuncture point), with 10 needles and electrical stimulation, in 90 patients and showed that upper endoscopy was much easier and better tolerated after real acupuncture. However, this study lacked a clearly defined primary endpoint measure and a detailed sample size calculation and had the disadvantage of using sham acupuncture instead of a real placebo acupuncture technique in the control group. Two additional, partially randomized studies have methodological limitations [19, 20]. An adequately designed, controlled clinical trial with a well-defined primary endpoint measure and detailed sample size calculation has not yet been conducted. Therefore, the objective of this trial was to compare the ability of real versus placebo acupuncture to improve tolerance of diagnostic esophagogastroduodenoscopy in patients not receiving intravenous sedation.

## Methods

The ACUPEND trial was designed as single-center, double-blinded, randomized superiority trial with a one-by-one allocation ratio into two parallel treatment arms. The study protocol was registered (Germanctr.de: DRKS00000164) and published in advance to ensure the transparency of the trial design and analysis procedures, after approval of the protocol by the Ethics Committee of the University of Heidelberg [21]. The trial was conducted in accordance with the 1989 Declaration of Helsinki [22] and the principles of Good Clinical Practice at the Interdisciplinary Center for Endoscopy of the University of Heidelberg. The study protocol was designed according to the Standards for reporting Interventions in Clinical Trials of Acupuncture (STRICTA) [23]. The statistical design and analysis were performed independently at the Institute of Medical Biometry and Informatics of the University of Heidelberg. There were no changes or amendments to the trial protocol throughout the study.

### Participants

All patients scheduled for elective diagnostic esophagogastroduodenoscopy in the Interdisciplinary Center for Endoscopy of the University of Heidelberg were screened and informed about the trial in detail by a trial investigator before their informed consent was requested. All patients older than 18 years and scheduled for an elective diagnostic esophagogastroduodenoscopy who refused systemic sedation were considered for participation. The exclusion criteria were refusal to participate, ASA score V, participation in another trial that could interfere with the primary endpoint, impaired mental state, expected lack of compliance, need for systemic sedation, emergency procedures, pregnancy, and known allergy to lidocaine anesthetic spray or acupuncture needle material.

### Randomization and intervention

To achieve comparable groups for known and unknown risk factors, randomization was performed using block randomization in a 1:1 allocation ratio. The random allocation sequence was generated by the Institute of Medical Biometry and Informatics with SAS version 9.1 (PROC PLAN). Treatment group allocation was performed using sealed and consecutively numbered opaque envelopes produced by the Institute of Medical Biometry and Informatics.

Patients were randomly sorted into groups after providing the investigator with informed consent; afterwards, the investigator performed the placebo or real acupuncture according to the randomization result.

Patients were prepared by staff nurses for the esophagogastroduodenoscopy as usual. A study nurse or investigator was present to monitor and document all procedures. All patients were positioned on a stretcher lying on their backs in a 30° reverse Trendelenburg position. Oxygen saturation and blood pressure were monitored by standard, non-invasive means. Staff personnel provided pharyngeal anesthesia using a topical xylocaine spray (AstraZeneca, Germany) for all patients. Before the endoscopic procedure was performed, the acupuncturist opened the allocation envelope. The physician performing the esophagogastroduodenoscopy and the assistant nurse were not informed of the allocation and entered the examination room after the acupuncture procedure was finished. Therefore, patients and examiners were both blinded to the trial intervention.

Only physicians with experience of at least 30 acupuncture procedures performed the study treatments. For further support, and to minimize any treatment bias, an acupuncture point search device (Silberbauer PS 3, Austria) was used.

### Control group: placebo acupuncture

**Fig. 1:**
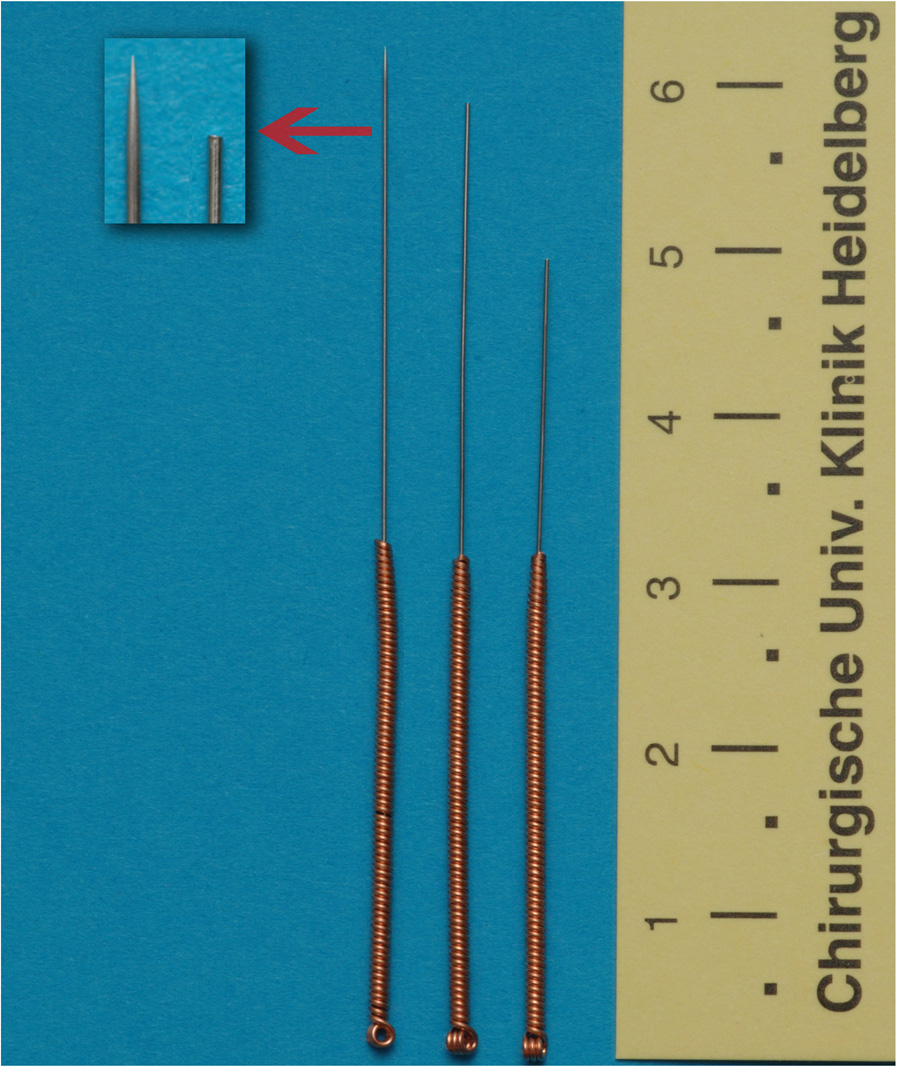
Placebo needle system

All control group patients received placebo acupuncture at the following points according to the Streitberger placebo acupuncture needle system procedure: Sinarteria respondens (Rs) 24 Chengjiang middle line to reduce choking [24], Pericardium (Pc) 6 Neiguan bilateral to reduce gastroenteral motility [25] and large intestine (IC) 4 Hegu bilateral to reduce nausea and vomiting [26] (Fig. 1).

**Fig. 2:**
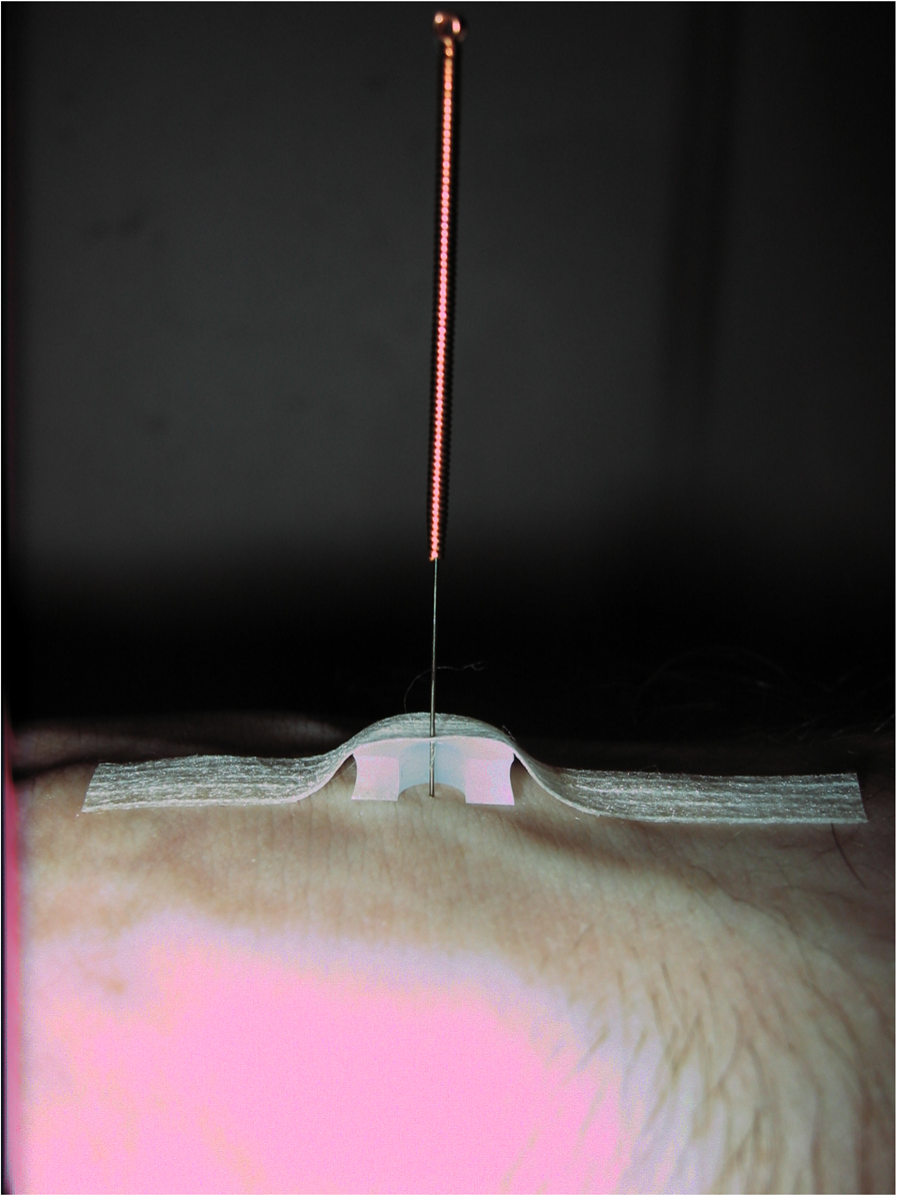
Cross-section of placed 32G placebo needle with synthetic ring and patch

After localizing the correct acupuncture point, supported by the search device, a synthetic ring (9 mm external diameter, 4 mm internal diameter, 3 mm height; Asiamed company, Munich, Germany) was fixed by a patch on the skin just above the selected point. Through this patched ring, a 32G placebo needle (No. PL, 30 × 0.3 mm stainless steel needle from the Asiamed company) was inserted for half to one inch. Because of the telescopic effect and the blunt tip of the placebo needle, an impression of penetration for the patient was imitated. All needles were placed and left in position for 5 min prior to and throughout the endoscopic procedure (Fig. 2).

### Experimental group: real acupuncture

After being prepared as described above, experimental group patients received a real acupuncture in accordance with the traditional Chinese medicine median theory at the following acupuncture points: Sinarteria respondens (Rs) 24 Chengjiang middle line to reduce choking, Pericardium (Pc) 6 Neiguan bilateral to reduce gastroenteral motility and large intestine (IC) 4 Hegu bilateral to reduce nausea and vomiting.

**Fig. 3:**
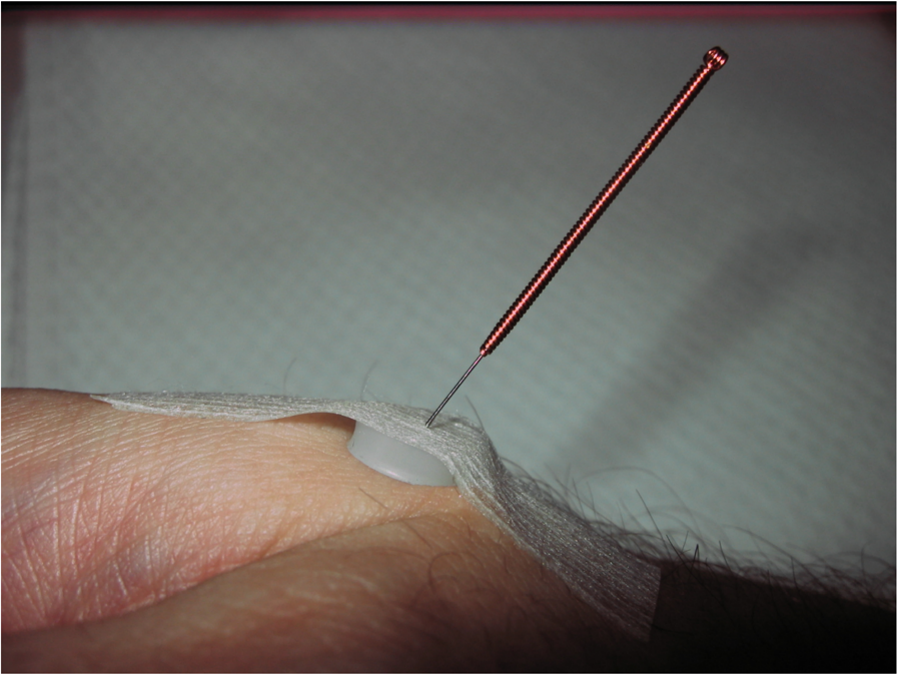
Real acupuncture: 32G needle in place with synthetic ring and patch

After localizing the correct acupuncture point supported by the search device, a synthetic ring (9 mm external diameter, 4 mm internal diameter, 3 mm height; Asiamed company) was fixed by a patch on the skin just above the selected point. Through this patched ring a 32G verum needle (No. 016 Special, 30 × 0.3 mm stainless steel needle from the Asiamed company) was inserted for half to one inch (Fig. 3). All needles were placed and left in position for 5 min prior to and throughout the endoscopic procedure.

Both procedures lasted about 10 min, comparable to the time needed to prepare patients for a systemic sedation.

### Outcomes

The main endpoint measure was the frequency of successfully performed esophagogastroduodenoscopy as a function of the randomized intervention.

Successful esophagogastroduodenoscopy was defined in accordance with the definition of Abraham et al. [27] as a composite score of patient satisfaction, with the procedure assessed on a Likert scale (from 1 = acceptable to 5 = unacceptable), and quality of examination, as assessed by the examiner. Each anatomic area (esophagus, stomach, duodenum up to the second stage, and proximal stomach viewed in retro flexion) that was adequately viewed received a score of 1, while area that were inadequately viewed were scored 0; this produced a maximum score of 4 if all anatomic areas could be well visualized. An esophagogastroduodenoscopy was counted as successfully performed if the patient’s satisfaction was rated 1 or 2 on the Likert scale and the examination quality score was 4/4 [27–30].

The primary endpoint measure was assessed immediately after the elective diagnostic esophagogastroduodenoscopy was completed; the results of quality of examination were documented in a case report form with tick boxes by the physician who performed the examination. The patients were asked by a study nurse to rate their satisfaction with the examination after the completion of esophagogastroduodenoscopy, prior to being told the results of their procedure and prior to discharge from the recovery room.

Secondary endpoint measures were willingness to repeat the procedure, defined as readiness of the patient to repeat the examination under the same conditions; heart rate (beats per minute); blood pressure (mmHg), and oxygen saturation (percent) assessed before esophagogastroduodenoscopy, after passage of the larynx, and after removal of the endoscope; the duration of the examination (min) from insertion to removal of the endoscope; and all peri-interventional complications as described and defined in the protocol publication.

### Sample size calculation

The sample size calculation was based on the two-sided chi-square test for difference with respect to the primary endpoint. A review of the literature identified a randomized controlled trial from Abraham et al. with a group of 419 patients, which compared the rate of successful esophagogastroduodenoscopy with and without sedation [27]. In both groups, pharyngeal anesthesia was performed. This trial showed a successful examination rate of 46 % in the group without sedation. A successful examination was defined as a composite score of patient satisfaction with the procedure and quality of the examination, as assessed by the endoscopist. For our trial, we adopted the same endpoint definition to facilitate using the 46 % success frequency of the non-sedated group as the baseline for our sample size calculation. We believe that an increase of the success frequency by 15 %, or to ≥61 %, in the real acupuncture group would be clinically relevant and therefore could have a significant impact on clinical practice. To detect this difference with a type I error rate of 0.05 (two-sided) with 80 % power, a sample size of 173 evaluable patients per group was necessary (SAS 9.1 PROC POWER). The drop-out rate within the intervention was expected to be about 2 % overall. Therefore, the total number of patients needed to be randomized was 354.

### Statistical analysis

#### Confirmatory analysis

The null hypothesis was assessed by testing the intervention effect in a primary analysis using a two-sided chi-square test. In a secondary analysis, a binary logistic regression model that took into account the covariates ‘intervention’ (placebo or acupuncture), age (<65 or ≥65), sex, and smoking status (yes or no) was used. A two-sided type I error rate of 0.05 was applied to the primary and secondary analysis. Confirmatory analysis was primarily based on the full analysis set, which is consistent with the intention-to-treat principle, by including all patients who were randomized into the two groups. This approach reflects the idea that the study should correspond to the conditions in clinical practice as closely as possible.

The secondary variables were analyzed in a descriptive manner by tabulating the measures of the empiric distributions. According to the scale level of the variables, means, standard deviations, medians, first and third quartiles, and minimum and maximum or absolute and relative frequencies, respectively, are reported. Descriptive values of *P* for the corresponding statistical tests comparing the treatment groups and associated 95 % confidence intervals are given.

The homogeneity of the treatment groups was demonstrated descriptively using the demographic data and baseline values. All statistical analyses were performed using SAS® software, Version 9.1 (or higher) of the SAS System for Unix (SAS Institute Inc., Cary, NC, USA).

## Results

### Patient enrollment

**Fig. 4:**
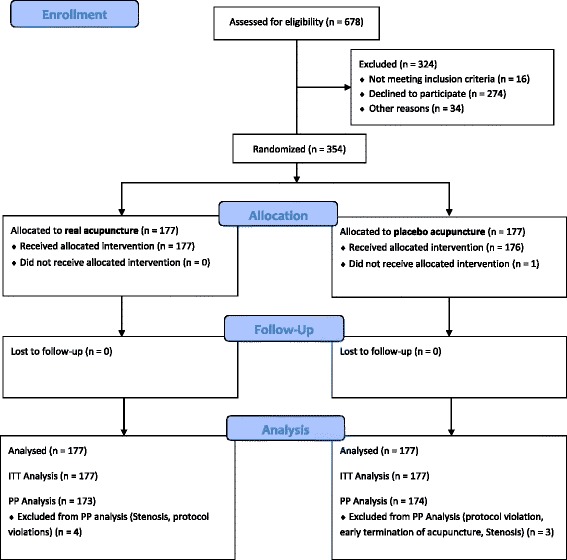
CONSORT Flowchart. *ITT* intention-to-treat; *PP* per-protocol

Out of 678 patients screened between February 2010 and July 2012, 354 were included (Fig. 4). The baseline characteristics of the two groups are listed in Table 1. Most of the evaluated parameters showed a similar distribution between both groups. Only the current smoking status was distributed unevenly. More current smokers were allocated to the placebo group.

**Table 1:** Baseline characteristics (intention-to-treat)

Characteristic		Placebo acupuncture group (*n* = 177)	Real acupuncture group (*n* = 177)	*P*
Sex (%)	Female	63 (35.6)	55 (31.1)	0.367^a^
Age (years)	Mean (± standard deviation)	53.4 (13.8)	52.3 (13.5)	0.422^b^
Body mass index (kg/m^2^)	Mean (± standard deviation)	26.7 (6.5)	25.8 (4.9)	0.139^b^
Current chemotherapy (%)	Yes	3 (1.7)	5 (2.8)	0.474^a^
Current smoking (%)	Yes	38 (21.5)	22 (12.4)	0.023^a^
ASA score (%)	I	32 (18.1)	30 (16.9)	0.768
	II	106 (59.9)	110 (62.1)	
	III	36 (20.3)	36 (20.3)	
	IV	3 (1.7)	1 (0.6)	

*ASA* American Society of Anesthesiology: Physical Status Classification System

*P* ≤ 0.05

^a^Chi squared test

^b^ *t* test

### Primary endpoint measure

The intention-to-treat analysis included 177 randomized patients in each group. Endoscopy could be performed successfully according to the definition by Abraham et al. [27] in 130 patients (73.5 %) in the real acupuncture group and in 129 patients (72.9 %) in the placebo group. No significant difference could be detected in univariate analysis with the chi-square test (*P* = 0.9045) or in the multivariate logistic regression model (odds ratio 0.929; 95 % confidence interval, 0.574–1.504). Only age showed a small significant difference favoring a successful endoscopy (odds ratio 1.023; 95 % confidence interval, 1.005–1.041) (Table 2).

**Table 2:** Multivariate logistic regression analysis of primary endpoint measure

Parameter	Odds ratio	95 % confidence interval	*P*
Intervention	0.929	0.574–1.504	0.7641
Smoking	0.933	0.498–1.747	0.8278
Age (years)	1.023	1.005–1.041	0.0114
Body mass index (kg/m^2^)	1.035	0.990–1.082	0.1324

### Secondary endpoint measures

The median duration of endoscopy was 7 min in both groups with a range of 2–20 min in the real acupuncture and 2–25 min in the placebo group (*P* = 0.406). Intravenous sedation was necessary in one patient in each group (*P* = 1). An acupuncture point search device was used in all but four (2.3 %) patients in the real acupuncture group and in all but two (1.1 %) patients in the placebo acupuncture group. Heart rate, blood pressure, and oxygen saturation showed no significant differences between the treatment groups at the beginning of the procedure, after passage of the larynx, or after removal of the endoscope. The gagging reflex was reduced in 98 (55.7 %) patients of the real acupuncture group and 94 (53.1 %) patients of the placebo acupuncture group (*P* = 0.627). A characteristic sensation due to manipulation of the penetrating needle (De Qi = sensation along the channel) was reported by 68 (38.6 %) patients in the real acupuncture group and 26 (14.7 %) patients in the placebo acupuncture group (*P* = 0.001). After completing the procedure, 153 (86.9 %) patients in the real acupuncture group and 155 (87.6 %) patients in the placebo acupuncture group were willing to repeat the procedure under the same conditions (*P* = 0.857).

No significant differences in peri-interventional complication frequencies were found between the intervention groups (Table 3).

**Table 3:** Frequency of peri-interventional complications

Parameter	Real acupuncture group	Placebo acupuncture group	Total	*P*
Hematoma	4 (2.3 %)	0 (0 %)	4 (1.1 %)	0.044
Bleeding	4 (2.3 %)	0 (0 %)	4 (1.1 %)	0.044
Nerve irritation	1 (0.6 %)	0 (0 %)	1 (0.3 %)	0.317
Bradycardia	1 (0.6 %)	0 (0 %)	1 (0.3 %)	0.317
Hypotension	1 (0.6 %)	0 (0 %)	1 (0.3 %)	0.317
Low oxygen saturation	1 (0.6 %)	0 (0 %)	1 (0.3 %)	0.317
Aspiration	0 (0 %)	0 (0 %)	0 (0 %)	–
Wound infection	0 (0 %)	0 (0 %)	0 (0 %)	–

## Discussion

The German sedation guideline states that sedation must be offered for every gastrointestinal endoscopy. However, standard use of systemic sedation during diagnostic esophagogastroduodenoscopy is responsible for nearly all severe complications of esophagogastroduodenoscopy [6] and for at least 40 % of the direct costs of the procedure [8]. In addition, indirect costs arise from patients losing a minimum of one day of work, so that significant economic advantages would accrue if the time of impairment could be shortened or eliminated [11].

Furthermore, patients who had an abdominal operation within a few days prior to esophagogastroduodenoscopy have an increased risk for delayed gastric empting and therefore an increased risk for aspiration during esophagogastroduodenoscopy, especially if sedation is given. Therefore, systemic sedation for these patients is often not possible, and general anesthesia is necessary if a procedure without sedation is not feasible. Therefore, it would be beneficial to have an effective alternative to systemic sedation.

A 2004 review of acupuncture in the context of gastrointestinal endoscopy concludes that acupuncture seems to be effective as a supportive intervention for gastrointestinal endoscopy, with outcomes similar to those of conventional premedication [17]. Concerning acupuncture during esophagogastroduodenoscopy, few studies have been published: one double-blind, controlled trial performed in 1978 and two partially randomized studies with methodological limitations in 1999 and 2002 [19, 20]. The randomized, controlled trial using real versus sham acupuncture (1 cm away from the acupuncture point) showed that upper endoscopy was much easier and better tolerated after real acupuncture [18]. However, a Cochrane review on acupuncture for migraine prophylaxis from 2009 reports that even acupuncture at the wrong place (sham acupuncture) can have a significant effect on the primary endpoint [15].

In our study, we decided to use Streitberger’s placebo needle system for the control group, simulating real acupuncture, instead of sham acupuncture, to avoid the necessity of penetrating of the skin [16]. In other trials, control groups have received obviously different treatments, and no attempt was made to evaluate the credibility of the placebo used, so that psychological factors might be largely responsible for differences between groups [31]. Streitberger et al. validated the placebo needle system successfully in two randomized controlled trials [16, 32]. The De Qi sensation sign was significantly different in our trial between intervention groups, but other trials have reported that the De Qi sign alone is not a valid predictor for the impact of the acupuncture effect [33].

In our study, we found no difference between the two acupuncture groups regarding successful esophagogastroduodenoscopy rate; our successful esophagogastroduodenoscopy rate was 73 % in both groups and thus nearly as high that of the sedation group (76 %) in Abraham’s study [27]. One possible interpretation of these results would be that the placebo needle system had the same effects as real acupuncture, because a small acupressure effect might have been induced by the method. Therefore, we decided that another control group without acupuncture would be necessary to clarify this problem. After approval of this planned prospective cohort study by the Ethics Committee of the University of Heidelberg, we included another 100 consecutive patients with the same inclusion and exclusion criteria as the ACUPEND trial. The rate of successful elective esophagogastroduodenoscopy was evaluated in this group in exactly the same manner as described in this paper.

This group without acupuncture had a 68 % successful esophagogastroduodenoscopy frequency, and therefore no significant difference in esophagogastroduodenoscopy success compared with the real or placebo acupuncture groups. Therefore, we assume that, in our study, neither real nor placebo acupuncture affected successful esophagogastroduodenoscopy frequency. However, a potential weakness of the present study is that this control group without acupuncture was not primarily planned in the study as a third arm, and patients were not randomized.

The willingness to repeat the procedure under the same conditions was 86.9 % versus 87.6 % in the real versus placebo acupuncture group. This rate is even higher than that of the sedated group in Abraham’s study (81 %) and much higher than in Abraham’s non-sedated group (61 %). Surprisingly, the highest willingness to repeat was in our non-sedated control group (94 %) [27]. This is even more astonishing, as all of our physicians performed our esophagogastroduodenoscopies; we did not exclude beginners. The difference in willingness might be due to cultural and social influences as important modifiers of patient satisfaction [26]. Waye noted the prevalent use of sedation in America (72 %) compared with Europe (56 %) and Asia (44 %) [34].

No evidence can be obtained in this trial concerning the specificity of acupuncture points; this requires another study where groups with different acupuncture points should be compared.

## Conclusions

Esophagogastroduodenoscopy without sedation is safe and can successfully be performed in two-thirds of patients.

Patients planned for elective esophagogastroduodenoscopy without sedation do not benefit from acupuncture of Sinarteria respondens (Rs) 24 Chengjiang middle line, Pericard (Pc) 6 Neiguan bilateral, and Dickdarm (IC) 4 Hegu bilateral, according to the traditional Chinese medicine meridian theory.
